# Preliminary Study on Different Types of Solid Dispersion Excipients for Improving the Water Solubility and Physical Stability of Celecoxib

**DOI:** 10.3390/pharmaceutics18030311

**Published:** 2026-02-28

**Authors:** Bin Liu, Shiqiao Rui, Yupan Cai, Ruoru Qian, Shuaipeng Feng, Zhu Liu, Qinfu Zhao

**Affiliations:** 1 Department of Pharmaceutics, School of Pharmacy, Shenyang Pharmaceutical University, 103 Wenhua Road, Shenyang 110016, China; 18341433302@163.com (B.L.); m18611538683_1@163.com (S.R.); gong1234561128@163.com (Y.C.); 13478025275@163.com (S.F.); liuzhu0899@163.com (Z.L.); 2 Changzhou Pharmaceutical Factory Co., Ltd., 518 East Laodong Road, Tianning District, Changzhou 213004, China; 13940107910@163.com

**Keywords:** dendritic mesoporous silica nanoparticles, commercial solid dispersion excipients, amorphous state, celecoxib, physical stability, bioavailability

## Abstract

The solubilization of poorly water-soluble drugs remains a critical challenge in pharmaceutical research. The formulation of solid dispersions employing mesoporous silica nanoparticles (MSN) constitutes a key strategy for enhancing the hydrophilicity and oral bioavailability of Biopharmaceutics Classification System (BCS) Class II drugs. Although several commercial mesoporous silica excipients have been approved for pharmaceutical use, there remains room for improvement regarding drug loading capacity, stability, and controllability of drug release. **Methods:** for this purpose, dendritic mesoporous silica nanoparticles (DMSN) with a radial dendritic structure and pH-responsive degradation properties were designed and synthesized using celecoxib (CEL) as the model drug, featuring a pore size of 21.51 nm. CEL was loaded onto DMSN and seven commercial solid dispersion excipients using the solvent evaporation method. **Results:** owing to its high surface area, pore volume, and radial structure, DMSN achieved 39.72% drug loading in an amorphous state, markedly improving wettability, dissolution, and physical stability. Accelerated stability tests showed that DMSN inhibited recrystallization, outperforming traditional solid dispersions. Pharmacokinetic studies in rats demonstrated that the oral bioavailability of CEL-DMSN was 1.29-fold higher than that of commercial celecoxib capsules. **Conclusions:** in conclusion, these results confirmed the potential of DMSN in enhancing the stability, promoting oral absorption, and reducing gastrointestinal irritation of poorly soluble drugs.

## 1. Introduction

According to FDA data, over 65% of drug candidate new chemical entities exhibit poor solubility, with water solubility being a core factor determining oral bioavailability [1,2]. Among these, Biopharmaceutics Classification System (BCS) Class II drugs—characterized by low solubility but good intestinal permeability—had become a key focus for formulation optimization [3]. From an economic perspective in drug development, solubility issues cause approximately 38% of candidate drugs to terminate development during the preclinical stage. Consequently, numerous compounds with potential therapeutic value cannot be translated into clinical drugs due to solubility limitations. Insufficient drug solubility thus poses a major challenge to both innovative drug development and the quality-cost profile of existing medicines, ultimately impeding pharmaceutical progress [4].

To address these challenges, solid dispersion technology has emerged as a cornerstone strategy [5]. By dispersing drugs in a high-energy state within hydrophilic carriers, this technology significantly improves the dissolution behavior of BCS Class II drugs [6]. Common carriers include polymers, surfactants, and mesoporous inorganic materials [7]. Traditional hydrophilic polymers (e.g., PVP, PEG) enhance wettability and inhibit crystallization, but they often suffer from low drug loading capacity, poor physical stability, and high hygroscopicity. Surfactants effectively dissolve drugs but may cause physiological irritation [8]. In comparison, mesoporous inorganic materials (especially mesoporous silica nanoparticles (MSN)) demonstrate unique potential for achieving efficient drug loading and stable dispersion due to their high specific surface area and ordered pore structures [9,10]. As an emerging carrier, MSN can restrict drug molecule migration through its nanopores, effectively delaying the recrystallization of amorphous drugs and thereby significantly enhancing the physical stability of formulations [11,12]. Compared to traditional MSN, dendritic mesoporous silica nanoparticles (DMSN) feature a radially arranged dendritic pore structure with larger pore size, higher specific surface area, and greater pore volume [13,14]. These characteristics not only enhance drug loading capacity but also facilitate faster molecular diffusion and more effective amorphous drug stabilization through spatial confinement. Recent studies have demonstrated the potential of DMSN in enhancing the oral bioavailability of poorly soluble drugs. Currently, several commercial MSN excipients have been approved for use [15]. However, systematic comparative studies between these commercial excipients and DMSN regarding drug loading capacity, dissolution performance, and stability remain insufficient [16,17]. Conducting such comparisons not only helps elucidate the intrinsic relationship between carrier structure and performance but also provides critical evidence for the rational design and carrier selection of oral delivery systems for poorly soluble drugs. Celecoxib (CEL) is a typical BCS Class II nonsteroidal anti-inflammatory drug (NSAID) widely used for treating arthritis and pain [18,19]. However, its poor water solubility, low oral bioavailability, and potential gastrointestinal irritation limit its clinical application [20,21]. Therefore, developing delivery systems that enhance the solubility of CEL, improve oral absorption, and reduce adverse reactions holds significant clinical importance.

**Scheme 1:**
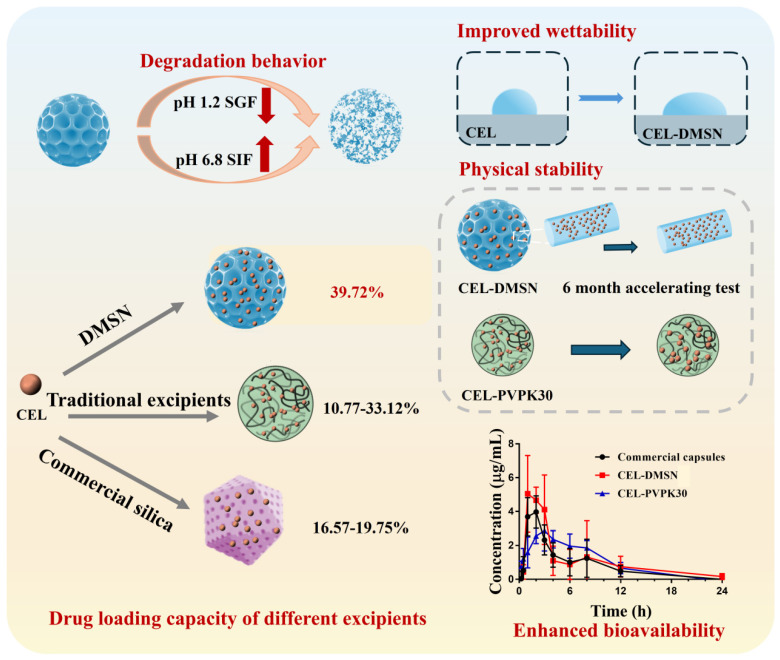
Dissolution mechanism and comparison of properties between DMSN and commercial excipients.

This study was designed and synthesized DMSN featuring branched radiating channels and pH-responsive rapid degradation properties, aiming to provide an “intelligent” solution for enhancing the intestinal absorption of BCS Class II drugs such as CEL. Previous studies have demonstrated the potential of DMSN for controlled drug delivery [14,22]; for instance, Liu et al. developed a dual-generation DMSN system capable of sequential release of ibuprofen and bovine serum albumin, enabling spatiotemporal control through hierarchical pore structures and chemical modification [23]. However, their work focused on dual-drug co-delivery, and systematic comparisons with commercial solid dispersion excipients in terms of drug loading capacity, physical stability, and in vivo performance for a single poorly water-soluble drug remain limited (Scheme 1). The performance of drug delivery systems constructed with DMSN was systematically compared against seven commercial solid dispersion excipients (PVP K30, PVP K90, TPGS, PEG4000, PEG6000, Syloid^®^XDP3050, and Syloid^®^244 FP) [24]. The evaluation criteria included drug loading capacity, amorphous state stability, in vitro dissolution, wettability, gastrointestinal safety, in vivo pharmacokinetics, and accelerated stability, aiming to fully assess its advantages as an oral delivery carrier. This study not only provides a novel strategy for overcoming oral absorption challenges of poorly soluble drugs but also offers theoretical support for rationally designing carriers with both high stability and excellent biocompatibility.

## 2. Materials and Methods

### 2.1. Materials

Celecoxib (CEL), Cetyltrimethylammonium bromide (CTAB), Sodium salicylate (NaSal, 99%), Tetraethyl orthosilicate (TEOS, 98%) and Triethanolamine (TEA, for High Performance Liquid Chromatography (HPLC)) were purchased from Aladdin Reagent Co., Ltd. (Shanghai, China). Syloid^®^XDP3050 and Syloid^®^244 FP were supplied from W.R. Grace & Co., Ltd. (Columbia, MD, USA). PVP K30, PVP K90, TPGS, PEG4000 and PEG6000 were supplied from BASF SE (Ludwigshafen, Germany). Over-the-counter celecoxib capsules were purchased from Fuyuan Pharmaceutical Co., Ltd. (Beijing, China), and according to the product information, their inactive ingredients include croscarmellose sodium, edible ink, gelatin, lactose monohydrate, magnesium stearate, povidone, sodium lauryl sulfate, and titanium dioxide.

### 2.2. Synthesis and Characterization of Carriers

#### 2.2.1. Synthesis, Characterization, and Degradation of DMSN

DMSN was synthesized via an anion-assisted soft template method [25]. TEA (68 mg) was mixed with water (25 mL) at 80 °C, followed by the addition of CTAB (380 mg) and NaSal (168 mg). After adding TEOS (4.0 mL) and reacting for 2 h, the product was collected, dried, and calcined at 550 °C for 6 h. The morphology and pore structure were characterized by transmission electron microscopy (TEM; TECNAI G2, FEI, Hillsboro, OR, USA) at an accelerating voltage of 200 kV. Scanning electron microscopy (SEM; ULTRA-PLUS, Zeiss, Oberkochen, Germany) was performed at 5 kV after the samples were sputter-coated with gold. Nitrogen adsorption–desorption isotherms were measured at 77 K using a specific surface area analyzer (BSD-660M, Beishide Instrument Technology, Beijing, China). The specific surface area was calculated by the Brunauer–Emmett–Teller (BET) method, and the pore size distribution was derived from the desorption branch of the isotherm using the Barrett–Joyner–Halenda (W_BJH_) model.

Degradation was simulated using a dialysis method. DMSN (10.0 mg) was placed in dialysis bags (0.8–1.4 kDa) with 2 mL of simulated gastric fluid (SGF, pH 1.2) or simulated intestinal fluid (SIF, pH 6.8), then immersed in 38 mL of the corresponding fluid at 37 °C. Quantitative analysis was performed using the molybdenum blue colorimetric method, and morphological evolution was tracked via TEM at 1, 3, 5, 7, and 14 days.

#### 2.2.2. Characterization of Commercial Excipients

The morphologies of all commercial excipients were observed via SEM. Wettability was assessed by measuring the contact angle on discs (8.5 mm diameter, compressed at 20 MPa for 30 s) using a contact angle goniometer (JC2000C1, Powereach, Shanghai, China). A 2 μL droplet of distilled water was deposited on the disc surface, and the angle was recorded after 10 s. Each sample was measured in triplicate.

### 2.3. Characterization of Amorphous Celecoxib (A-CEL)

A-CEL was prepared using the melt-quench method by heating CEL to 190 °C and rapidly cooling it with liquid nitrogen. Morphology and surface area were compared with crystalline CEL using SEM and N_2_ adsorption–desorption.

### 2.4. Construction and Characterization of Drug-Loading Systems

#### 2.4.1. Optimization of Drug-Loading Ratio

A solvent evaporation method was employed to construct the drug-loading system. Taking CEL-DMSN as an example, 20 mg of CEL was precisely weighed and placed in 3.00 mL of ethanol. The mixture was sonicated until complete dissolution, followed by the addition of 30 mg of DMSN. After ultrasonication for 0.5 h, the mixture was stirred until the solvent evaporated. The resulting sample underwent vacuum drying. Based on research findings regarding traditional carriers and preliminary experimental results, drug delivery systems with varying mass ratios were prepared. The optimal drug-to-carrier ratio for maintaining an amorphous state was determined by Differential Scanning Calorimetry (DSC, Mettler Toledo, Greifensee, Switzerland) and X-ray diffraction (XRD, Rigaku Corporation, Tokyo, Japan). For PEG4000 and PEG6000, due to their inherent crystallinity, the ratio was optimized through in vitro dissolution tests conducted using the USP Apparatus II (paddle method) with a ZRS-8G intelligent dissolution meter (Tianda Tianfa Technology Co., Ltd., Tianjin, China). The dissolution medium was 900 mL of a 0.2% sodium dodecyl sulfate (SDS) solution, maintained at a temperature of 37.0 °C. The dissolution vessels were standard 1 L round-bottom glass USP dissolution vessels, and the paddles were rotated at 100 rpm. Carriers containing an amount equivalent to 3 mg of CEL were precisely weighed and introduced into the dissolution vessels. Samples of 5 mL were collected at 10, 20, 30, 45, and 60 min, and their absorbance was measured at a wavelength of 242 nm. According to calculations, the drug dosage was much lower than the amount required to reach saturation in the dissolution medium (11.7 mg). Dissolution methodology is in the Supporting Information.

#### 2.4.2. Morphology, Structure, and Loading Capacity

The final systems were observed via SEM. Nitrogen adsorption was used to verify CEL loading within the nanopores by measuring changes in surface area and pore volume. Drug loading (DL%) was quantified by UV spectrophotometry (UV-1800, Shimadzu, Kyoto, Japan) at 242 nm.

### 2.5. Evaluation of Physicochemical Properties

#### 2.5.1. Wettability and Flowability

Contact angles were measured on compressed discs to evaluate hydrophilicity. Flowability was evaluated via the determination of the angle of repose with the fixed funnel method. Briefly, the sample was freely dropped onto a horizontal plate through a standard funnel to form a conical pile. The diameter of the bottom of the pile and the height of the funnel were measured to calculate the angle of repose (θ), and its flowability was determined by θ.

#### 2.5.2. In Vitro Dissolution

Dissolution tests were performed for the raw drug, all drug delivery systems, and commercial celecoxib capsules. Based on these results, CEL-PVP K30 and CEL-DMSN were selected for subsequent evaluations.

### 2.6. Gastric Irritation Study

SD rats (n = 3/group) were administered saline, CEL-PVP K30, CEL capsules, or CEL-DMSN (5.20 mg/kg) daily for 7 days. Collect gastric tissue samples, fix them with paraformaldehyde, embed them in paraffin, slice them, and perform histopathological evaluation using hematoxylin and eosin (H&E) staining.

### 2.7. In Vivo Pharmacokinetics

SD rats (n = 5/group) were given oral doses (5.20 mg/kg) of the selected formulations. Blood samples were collected at intervals from 0.5 to 24 h and analyzed via HPLC to determine pharmacokinetic parameters. In vivo pharmacokinetic methodology is in the Supporting Information.

### 2.8. Accelerated Stability Study

Samples were stored at 40 °C/75% RH for 6 months. At months 0, 1, 2, 3, and 6, stability was assessed through visual inspection, content uniformity, DSC (for amorphous state), and dissolution performance.

### 2.9. Statistical Methods

Quantitative data were expressed as mean ± standard deviation (SD). For multiple-group comparisons, a one-way analysis of variance (ANOVA) was performed. Dunnett’s test was used for comparisons against a control group, while Tukey’s honestly significant difference (HSD) test was used for pairwise comparisons among other groups. Statistical significance was set at * *p* < 0.05, ** *p* < 0.01, and *** *p* < 0.001; ns (not significant) indicates *p* ≥ 0.05.

## 3. Results and Discussion

### 3.1. Comparison of Structure and Properties of DMSN and Commercial Excipients

#### 3.1.1. Characterization and In Vitro Degradation of DMSN

**Figure 1:**
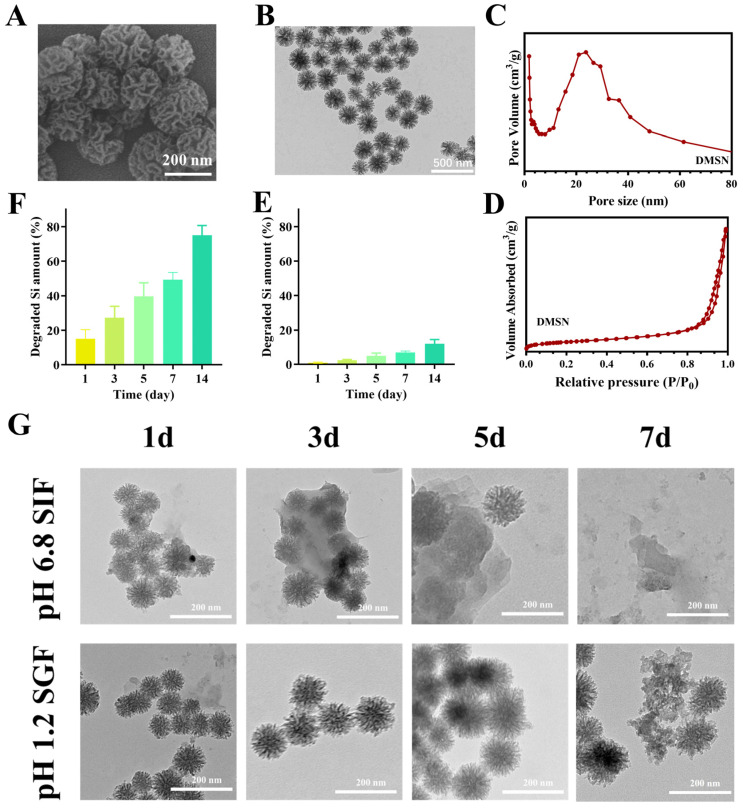
(**A**) SEM image of DMSN. (**B**) TEM image of DMSN. (**C**) Pore size distribution curves of DMSN. (**D**) N_2_ adsorption/desorption isotherms of DMSN. (**E**) DMSN degradation product quantification under pH 1.2 SGF conditions (n = 3). (**F**) DMSN degradation product quantification under pH 6.8 SGF conditions (n = 3). (**G**). TEM of DMSN degradation residues in SGF (pH 1.2) and SIF (pH 6.8).

The specific surface area and pore volume of DMSN were critical factors influencing drug loading efficiency and release behavior [25]. Typically, an increased specific surface area made available a greater number of active sites for drug adsorption [26]. In this study, DMSN with spherical morphology was successfully prepared using an anion-assisted soft template method. As shown in Figure 1A,B, TEM characterization results revealed that the prepared DMSN exhibited a regular spherical shape with an average diameter of approximately 200 nm, featuring internal dendritic mesoporous channels radiating from the center. SEM images further showed abundant open pores and wrinkled hierarchical structures on the particle surface; this porous architecture facilitates rapid drug diffusion and efficient loading. As shown in Figure 1C,D, the nitrogen adsorption–desorption isotherms of DMSN exhibited a typical Type IV curve, characteristic of mesoporous materials. The N_2_ adsorption–desorption specific surface area (S_BET_), pore volume (V_p_), and pore size (W_BJH_) were calculated to be 649.87 m^2^/g, 3.49 cm^3^/g, and 21.51 nm, respectively.

The safety of DMSN has been confirmed through extensive in vitro and in vivo studies, primarily manifested in its good biocompatibility, degradability, and the safety of its degradation products [27,28,29]. For nanocarriers, in vivo degradation characteristics were closely related to biocompatibility [30]. In the biological environment, the degradation of DMSN is primarily attributed to the nucleophilic attack of OH^−^ ions, leading to the cleavage of Si–O–Si bonds. The dissolution products are silicic acid or polysilicic acid [31]. To preliminarily assess the in vivo degradation potential of the prepared DMSN, this study first employed an in vitro simulated degradation experiment combined with the molybdenum blue colorimetric method for quantitative analysis. In the in vitro degradation system, DMSN gradually decomposed and released silicate ions, which reacted with ammonium molybdate to form a silicomolybdic yellow intermediate. This was subsequently reduced to a silicomolybdic blue complex, the absorbance of which was proportional to the Si content, thereby allowing for the quantitative determination of dissolved silicon [32]. The in vitro degradation results are shown in Figure 1E,F. The degradation of DMSN was more rapid in SIF than in SGF. Specifically, the degradation amount of DMSN in SGF on the 1st day was only 1.03%, whereas in SIF, it reached 15.00%. By the 5th day, the degradation in SGF increased to 5.00%, while in SIF it reached 39.67%. On the 7th day, the degradation in SIF reached 39.33%, compared to only 7.00% in SGF. By the 14th day, degradation reached 69.67% in SIF and only 12% in SGF. The cumulative amount of DMSN degradation produced increased in a time-dependent manner.

The morphology of DMSN during degradation in SGF and SIF was observed to understand its degradation characteristics. As shown in Figure 1G, the DMSN structure remained intact in SGF. In SIF, however, obvious cracks appeared in the DMSN channels on the 1st day; by the 3rd day, the characteristic structure of the carrier gradually disappeared; and by the 7th day, the dendritic structure of DMSN was completely destroyed. This morphological evolution was highly consistent with the quantitative results from the molybdenum blue colorimetric method, further confirming the rapid degradation characteristics of DMSN in the intestinal environment. The significant difference in degradation efficiency between the two simulated fluids primarily stemmed from their different pH environments and ionic compositions. SGF was strongly acidic; in this environment, the surface silanol groups of DMSN were easily protonated to form a stable structure, inhibiting the hydrolysis of siloxane bonds. SIF was weakly alkaline and contained high concentrations of anions such as HCO_3_^−^ and HPO_4_^2−^. The weakly alkaline environment promoted the deprotonation of surface silanol groups to form more nucleophilic -SiO^−^ species. Additionally, anions could bind to silicon atoms through coordination, further accelerating the hydrolytic cleavage of Si-O-Si bonds, ultimately leading to higher degradation efficiency of DMSN in SIF [12].

#### 3.1.2. Characterization of Commercial Excipients

**Figure 2:**
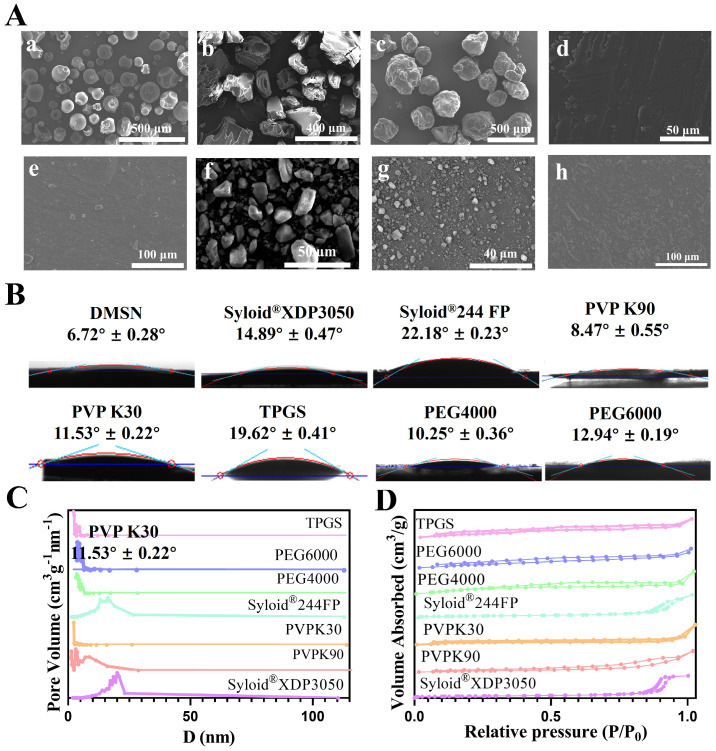
(**A**) SEM image of (**a**) PVP K30, (**b**) PVP K90, (**c**) TPGS, (**d**) PEG 4000, (**e**) and PEG 6000. (**f**) Syloid^®^XDP3050. (**g**) Syloid^®^244 FP. (**h**) CEL. (**B**) Contact angle of DMSN, Syloid^®^XDP3050, Syloid^®^244 FP, PVP K90, PVP K30, TPGS, PEG4000 and PEG6000. (**C**) Pore size distribution curves of commercial excipients. (**D**) N_2_ adsorption/desorption isotherms of commercial excipients.

**Figure 3:**
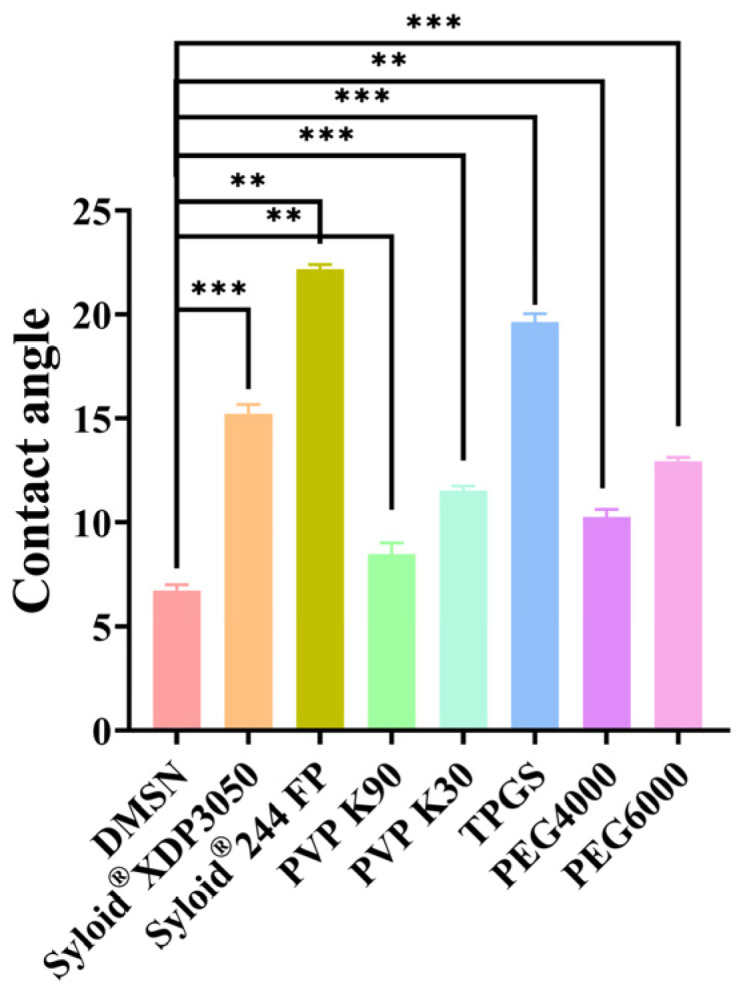
Column chart of contact angle for DMSN, Syloid^®^XDP3050, Syloid^®^244 FP, PVP K90, PVP K30, TPGS, PEG4000 and PEG6000 (** *p* < 0.01, *** *p* < 0.001).

SEM characterization results showed significant differences in the morphology of different commercial excipients. As shown in Figure 2A(a–g), PVP K30 presented as sphere-like particles, while PVP K90 and TPGS appeared as irregular blocky forms. PEG4000 and PEG6000 exhibited flake-like structures. The pharmaceutical mesoporous silica excipient Syloid^®^XDP3050 presented a “pomegranate seed-like” polygonal structure, and Syloid^®^244 FP appeared as irregular spheres. As shown in Figure 2C,D, and summarized in Table 1 for S_BET_ and other data comparisons, each carrier possessed unique physical properties. Among the eight carriers, DMSN showed better uniformity, and its S_BET_, V_P,_ and W_BJH_ values were superior to those of other commercial excipients, indicating stronger potential for drug loading. With the data following a normal distribution and homogeneity of variance, the contact angle measurement results (Figure 2B and Figure 3) showed that DMSN (6.72° ± 0.28°) had a significantly lower contact angle than Syloid^®^XDP3050 (14.89° ± 0.47°) (*p* < 0.001), demonstrating its superior hydrophilicity. This enhanced hydrophilicity thus improved the wetting and dispersion of hydrophobic drugs. These results indicated that DMSN exhibited excellent structural properties and hydrophilicity, showing promising prospects as an efficient drug carrier.

### 3.2. Evaluation of Amorphous Celecoxib

**Figure 4:**
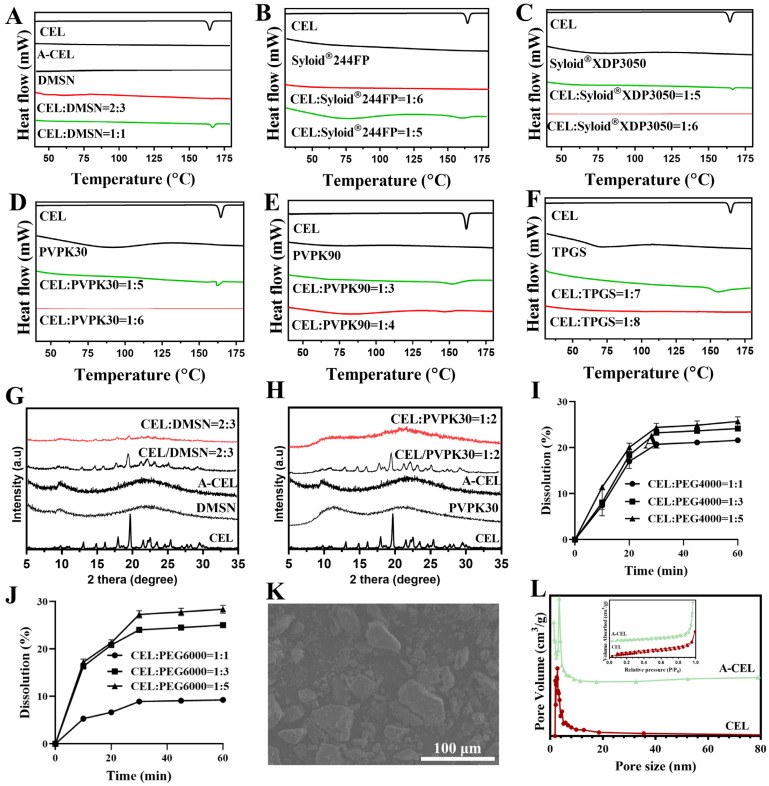
The DSC curves of (**A**) CEL-DMSN, (**B**) CEL-Syloid^®^244 FP, (**C**) CEL-Syloid^®^XDP3050, (**D**) CEL-PVP K30, (**E**) CEL-PVP K90 and (**F**) CEL-TPGS. The XRD patterns of (**G**) CEL-DMSN and (**H**) CEL-PVP K30. Dissolution behaviors of (**I**) CEL-PEG4000 in 0.2%SDS and (**J**) CEL-PEG6000 in 0.2%SDS. (**K**) SEM images of A-CEL. (**L**) Pore size distribution curves and N_2_ adsorption/desorption isotherms of A-CEL and CEL.

To demonstrate the stabilizing effect of the carrier on amorphous drugs, preparations of amorphous drugs were conducted. A-CEL was prepared using the melt-quench method. SEM comparison of crystalline CEL and A-CEL (Figure 2A(h) and Figure 4K) showed that crystalline CEL particles were relatively regular in shape with a concentrated particle size distribution and smooth, passivated edges. In contrast, A-CEL appeared as irregular blocky aggregates with a broad particle size distribution and high surface roughness, indicating significant differences in micromorphology. N_2_ adsorption–desorption analysis (Figure 4L) showed that compared to A-CEL, CEL had a relatively smaller specific surface area S_BET_ = 2.69 m^2^/g vs. 3.59 m^2^/g for A-CEL). The pore sizes W_BJH_ were 10.25 nm and 38.65 nm, respectively. Theoretically, a larger specific surface area increased the contact area between the drug and the dissolution medium, which was beneficial for improving the dissolution rate and extent of BCS Class II drugs, providing a physical basis for further optimization of the drug-loaded system.

### 3.3. Construction and Characterization of Drug-Loaded Systems

#### 3.3.1. Determination of Optimal Drug Loading Ratio

In the solvent evaporation process, the drug-to-carrier mass ratio directly determined the resultant physical state of the drug [33]. If the drug input exceeded the carrier’s loading capacity, excess drug may precipitate as crystals on the carrier surface. To determine the optimal ratio that maximizes the maintenance of the amorphous state, DSC analysis was performed on systems with different ratios. As shown in Figure 4A–F, when the mass ratios were CEL:DMSN = 2:3; CEL:Syloid^®^244 FP = 1:6;Syloid^®^XDP3050 = 1:6; CEL:PVP K30 = 1:2; and CEL:TPGS = 1:8, the DSC curves showed no obvious drug melting peaks, indicating that the drug was primarily dispersed in an amorphous state within the carrier pores or matrix at these ratios [34]. These ratios were thus determined as the optimal drug loading ratios for the respective carriers.

For crystalline drugs, XRD patterns exhibited distinct characteristic peaks, whereas amorphous samples present broad, featureless humps. As shown in Figure 4G,H, crystalline CEL raw material showed characteristic diffraction peaks at 2θ values of 13.06°, 14.88°, 16.12°, 17.96°, 19.68°, 21.52°, 22.50°, and 23.48°. Analysis of physical mixtures revealed crystal diffraction peaks, whereas the drug carriers did not exhibit such features. The CEL-loaded systems at the ratios optimized by DSC exhibited no detectable crystalline diffraction peaks, confirming the amorphous dispersion of the drug within the carrier at these specific formulations.

While the optimal ratios for six of the drug-loaded systems could be screened by combining the absence of melting peaks in DSC and characteristic peaks in XRD, PEG solid dispersions possess inherent crystallinity. The determination of the optimal ratio for PEG4000 and PEG6000 was limited when using DSC and XRD alone and required analysis via other methods. Additionally, PEG series carriers exhibited a characteristic where higher drug loading correlates with poorer wettability, thereby inhibiting drug dissolution. Therefore, in vitro dissolution experiments were employed to screen and evaluate PEG4000 and PEG6000 formulations with different ratios. SDS was an anionic surfactant commonly used for dissolution testing of poorly water-soluble drugs (BCS Class II) to maintain sink conditions. At a concentration of 0.2% (*w*/*v*), SDS effectively simulated the solubilization environment of the gastrointestinal tract and played an important role in improving drug solubility [35]. As shown in Figure 4I,J, in 0.2% SDS medium, the dissolution rate of PEG4000 systems with different mass ratios was above 20%, with the 1:5 ratio showing the best performance. The PEG6000 system at a 1:5 ratio also showed a clear advantage over other ratios. Consequently, a mass ratio of 1:5 was selected as the optimal drug loading ratio for both PEG4000 and PEG6000.

#### 3.3.2. Morphological and Structural Analysis of Drug-Loaded Systems

**Figure 5:**
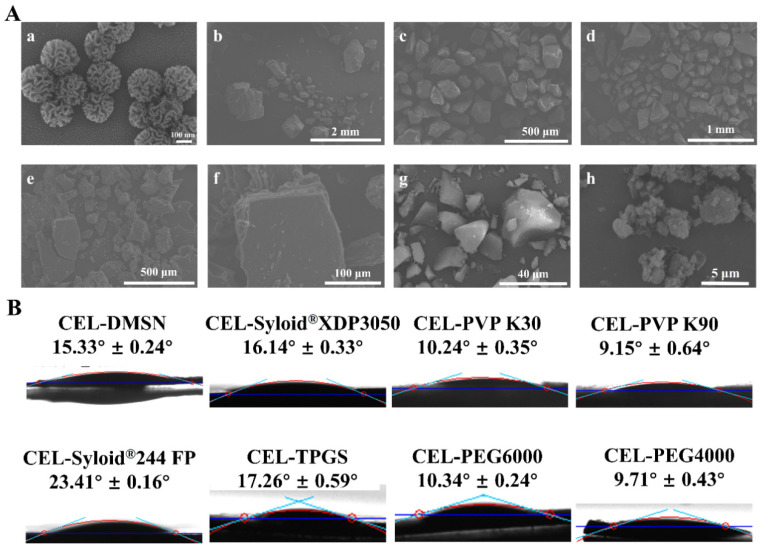
(**A**) SEM images of (**a**) CEL-DMSN, (**b**) CEL-PVP K30, (**c**) CEL-PVP K90, (**d**) CEL-TPGS, (**e**) CEL-PEG4000, (**f**) CEL-PEG6000, (**g**) CEL-Syloid^®^XDP3050 and (**h**) CEL-Syloid^®^244 FP. (**B**) Contact angle of CEL-DMSN, CEL-Syloid^®^XDP3050, CEL-Syloid^®^244 FP, CEL-PVP K90, CEL-PVP K30, CEL-TPGS, CEL-PEG4000 and CEL-PEG6000.

SEM systematic observation of the eight drug-loaded systems (Figure 5A(a–h)) revealed that CEL-DMSN maintained a dendritic spherical morphology with abundant irregular pores on the surface, and the particle size did not change significantly after drug loading. CEL-PVP K30 appeared as blocky structures with a wide particle size range, while CEL-PVP K90 and CEL-TPGS were relatively uniform. CEL-PEG4000 and CEL-PEG6000 showed sphere-like and blocky shapes. CEL-Syloid^®^XDP3050 presented a blocky polygonal structure, and CEL-Syloid^®^244 FP appeared as irregular spheres. The structural characteristics of the carrier directly influenced morphology and performance after drug loading; mesoporous silica was particularly conducive to efficient drug loading and release due to its significant specific surface area and ordered porous structure.

To reveal the intrinsic relationship between carrier pore characteristics and drug loading, N_2_ adsorption–desorption was used to systematically characterize the S_BET_, V_P_, and W_BJH_ distributions of the drug-loaded systems. Analysis of the parameter changes before and after drug loading (Table 2) showed that, except for the PVP series and TPGS systems, the specific surface area, pore volume, and average pore size of the other systems significantly decreased compared to the blank carriers, with the synthesized DMSN showing the greatest reduction in specific surface area. This phenomenon indicated that CEL molecules entered the nanopores of the carrier through physical interactions, occupying the internal pore space and reducing the effective pore volume and size, further confirming the formation of stable interactions between the drug and the carrier.

#### 3.3.3. Drug Loading Capacity

The actual drug loading capacities of the eight different systems determined by UV spectrophotometry were shown in Table 2. The synthesized DMSN system achieved the highest drug loading, reaching 39.72%. Among commercial excipients, PVP K90 and PVP K30 systems had relatively high loading, while others ranged between 14% and 20%. This was likely related to their smaller pore sizes, which were unfavorable for the uniform distribution of the drug within the channels. These results further confirmed that the structural characteristics of the carrier directly influenced its drug loading performance [36,37].

### 3.4. Physicochemical Properties Evaluation

#### 3.4.1. Wettability

**Figure 6:**
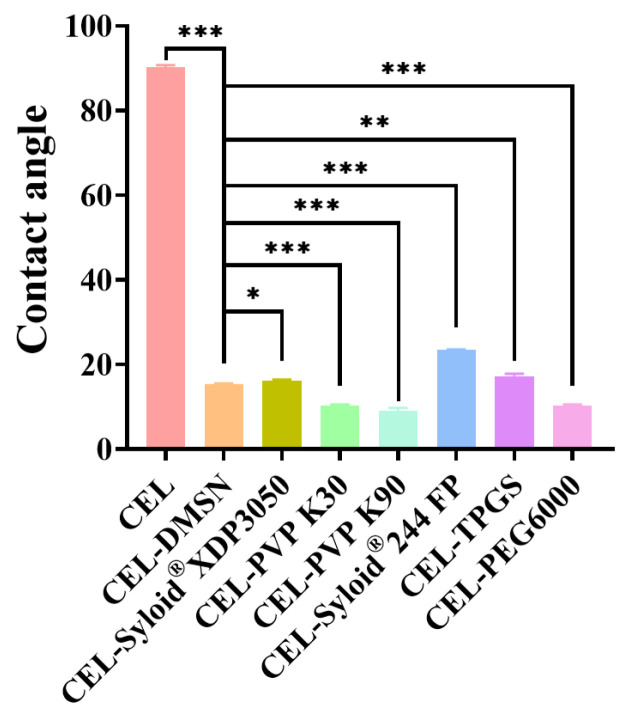
Column chart of contact angle for CEL, CEL-DMSN, CEL-Syloid^®^XDP3050, CEL-PVP K30, CEL-PVP K90, CEL-Syloid^®^244 FP, CEL-TPGS, and CEL-PEG6000 (* *p* < 0.05, ** *p* < 0.01, *** *p* < 0.001).

To systematically evaluate the effect of different carriers on improving drug wettability, contact angle measurements were analyzed (Figure 5B and Figure 6 and Table 3). The results showed that the contact angle of raw CEL was 90.25°, exhibiting the strong hydrophobicity typical of poorly soluble drugs. After loading into the eight carriers, wettability significantly improved. The contact angle of CEL-DMSN dropped to 15.33°, achieving a superhydrophilic state (θ < 30°). The high specific surface area and abundant surface hydroxyl groups of DMSN contributed to this improvement, likely by enhancing drug dispersion and reducing solid–liquid interfacial tension through hydrogen bonding. This also explained the significant improvement in wettability for Syloid^®^XDP3050 and Syloid^®^244 FP systems. Additionally, the PVP series systems had contact angles around 10°, with hydrophilicity mainly derived from the hydrogen bond network formed between the alcohol/amide groups in the molecular chain and water. The PEG series also exhibited excellent wettability, with hydrophilic groups such as -OH enhancing interactions with water and promoting the spreading of water molecules on the drug surface. This study revealed the contribution by which different carriers improved drug solubility from the perspective of wettability, providing a theoretical basis for enhancing the dissolution rate and bioavailability of oral formulations.

#### 3.4.2. Flowability

The angle of repose measurement was employed to systematically assess how drug loading influenced the flowability of the carrier powders. Data from Table 3 indicated that for most carriers, the angle of repose did not change significantly before and after drug loading, suggesting a limited impact on flowability. Specifically, the angle of repose for the DMSN system after drug loading was 29.64°, slightly lower than the pre-loading value of 30.97°, indicating good flowability. The results showed that the powder flowability of the seven commercial excipients after drug loading met production requirements, with no significant changes in the angle of repose. Therefore, selecting appropriate carriers and drug loading amounts was crucial for maintaining good flowability.

#### 3.4.3. In Vitro Dissolution Behavior

**Figure 7:**
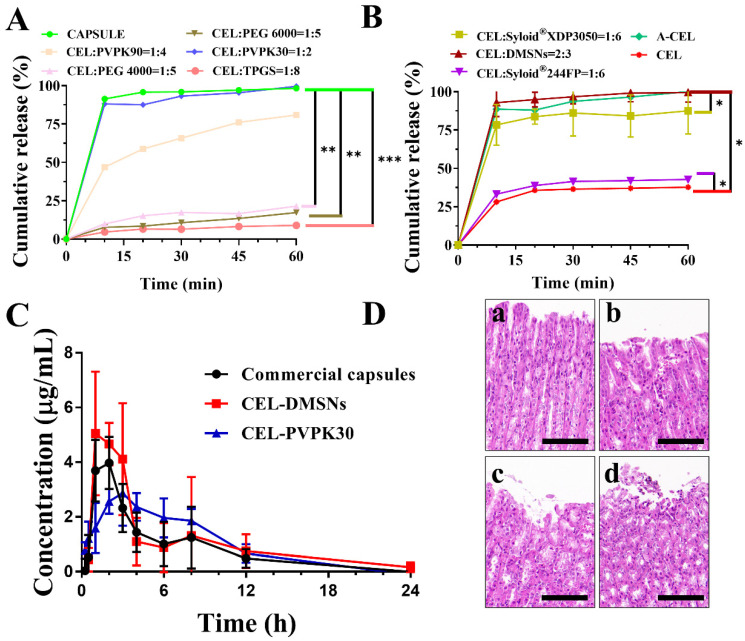
(**A**,**B**) Dissolution behaviors of drug delivery systems. (**C**) Plasma concentration–time curves of CEL following oral administration of CEL-DMSN, commercial capsules and CEL-PVP K30 (n = 5). (**D**) Assessment of rat gastric mucosal irritation following one-week oral administration of (**a**) saline, (**b**) CEL-DMSN, (**c**) CEL-PVP K30, and (**d**) commercial CEL capsules (Scale bar = 100 µm, n = 3), (* *p* < 0.05, ** *p* < 0.01, *** *p* < 0.001).

The dissolution profiles of raw CEL, different drug-loaded systems, and celecoxib capsules were compared (Figure 7A,B) to systematically evaluate the promotion of drug release by each system. The synthesized DMSN system excelled in enhancing the drug dissolution rate. Within 20 min, the cumulative dissolution rate of CEL-DMSN reached over 90%, significantly higher than that of other commercial excipients. This was mainly attributed to the high specific surface area, large pore volume, and open mesoporous channels of DMSN, which provided a favorable diffusion space for drug release. Among commercial excipients, PVP K30 showed the highest cumulative drug dissolution, while TPGS showed relatively lower performance, highlighting the critical impact of carrier structure on drug release behavior. Specifically, the high dissolution of PVP K30 may be closely related to its higher drug loading capacity and unique structural features. Notably, CEL was in an amorphous form in the drug-loaded systems, which significantly increased drug solubility and accelerated the dissolution process. Therefore, commercial celecoxib capsules, CEL-PVP K30, and CEL-DMSN were selected for further investigation.

### 3.5. Evaluation of Gastric Mucosal Irritation

To evaluate the safety of the oral drug-loaded systems, HE staining results of rat gastric mucosa were observed as shown in Figure 7D(a–d). The saline group showed intact gastric mucosal epithelium, no hyperemia or edema in the lamina propria, no inflammatory cell infiltration, and normal mucosal structure. The celecoxib capsule group showed mild hyperemia in the lamina propria and a small amount of inflammatory cell infiltration. The CEL-PVP K30 group showed slightly less hyperemia than the capsule group. The CEL-DMSN group exhibited only very slight tissue hyperemia and no significant inflammatory cell infiltration, showing the lowest degree of hyperemia among all groups. During the experiment, no abnormal behaviors (e.g., lethargy, loss of appetite) or deaths were observed in rats, further supporting the favorable gastrointestinal compatibility of the CEL-DMSN system. The reduction in gastric irritation may be attributed to the entrapment of CEL within the mesoporous structure of DMSN, which limits its direct exposure to the gastric mucosa. However, given the limited sample size (n = 3), these findings are preliminary, and further studies with larger sample sizes are warranted to confirm the gastrointestinal safety of the formulation.

### 3.6. In Vivo Pharmacokinetics

The dissolution process was the rate-limiting step for the absorption of BCS Class II drugs; enhancing dissolution properties could effectively improve bioavailability [38]. This study systematically evaluated the effect of different drug-loaded systems on the oral bioavailability of CEL via pharmacokinetic experiments. The mean plasma concentration–time curves of rats after oral administration of CEL were plotted in Figure 7C, and relevant pharmacokinetic parameters were summarized in Table 4. The oral bioavailability of CEL-DMSN and CEL-PVP K30 was 1.29 and 1.19 times that of commercial celecoxib capsules, respectively. Notably, the CEL-DMSN group exhibited the highest C_max_ value of (6.16 ± 1.47) μg/mL, which was significantly different from that of the CEL-PVP K30 group (3.14 ± 0.88) μg/mL (** *p* < 0.01). The CEL-DMSN group exhibited the shortest T_max_ value, suggesting accelerated drug absorption kinetics. This characteristic aligned with the pharmacokinetic profile typical of immediate-release formulations. These results may be explained by two main factors: (i) the amorphous dispersion of CEL within DMSN, which enhances its apparent solubility and dissolution rate; and (ii) the mesoporous structure of DMSN, which offers a high specific surface area and short diffusion pathways, facilitating rapid drug release and subsequent absorption. These characteristics collectively promoted the dissolution and absorption process of CEL, significantly increasing its oral bioavailability [39].

### 3.7. Accelerated Stability Evaluation

Highly dispersed amorphous drugs exist in a high-energy metastable state and tend to recrystallize during storage (especially under the influence of temperature and humidity). The dissolution advantage was abolished following drug recrystallization. Visual inspection involved observing physical changes in samples under accelerated test conditions, providing a basis for evaluating sample quality and serving as a key reference for ensuring safety and stability throughout the experiment. Appearance examination of A-CEL, CEL-DMSN, and CEL-PVP K30 samples at 0, 1, 2, 3, and 6 months showed no significant changes in morphology. This indicated that storage under accelerated conditions for 6 months did not alter the physical appearance of these samples.

As shown in Table S5, the CEL-DMSN system demonstrated excellent stability under accelerated conditions, with drug loading decreasing by only 1.7% over 6 months and content fluctuations remaining below 3.0% at all time points. In contrast, the CEL-PVP K30 system showed poorer stability, with a 6.0% decrease in drug loading and significantly increased fluctuations in the later stages, primarily due to the hygroscopicity of PVP. A-CEL exhibited the least ideal stability, with drug loading decreasing by 4.3% in the first month and remaining significantly lower than the initial value at 6 months.

**Figure 8:**
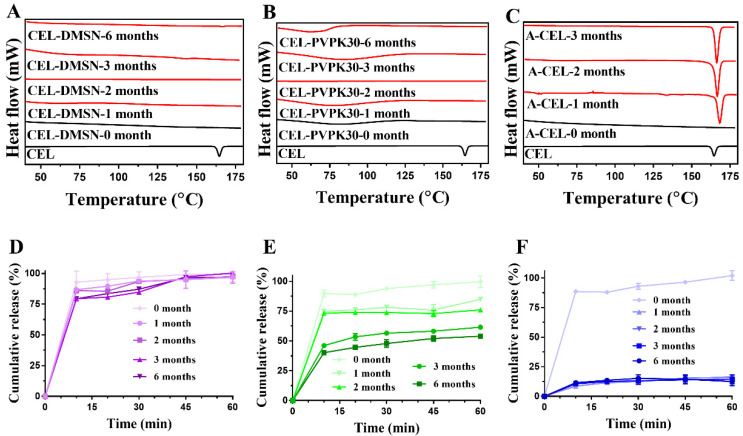
Representative DSC curves of (**A**) CEL-DMSN, (**B**) CEL-PVP K30, and (**C**) A-CEL obtained from accelerated stability testing. Dissolution behaviors of CEL-DMSN (**D**), CEL-PVP K30 (**E**), and A-CEL (**F**) under accelerated conditions (n = 3).

As shown in Figure 8A–C, DSC characterization results indicated significant differences in the stability of the amorphous state among the three systems under accelerated conditions. A-CEL showed an obvious crystallization exothermic peak in the first month, indicating a rapid transition to the crystalline state, whereas the crystallization signals for CEL-DMSN and CEL-PVP K30 were significantly weaker. CEL-DMSN possessed a greater advantage in maintaining the amorphous state due to the spatial confinement effect of its mesoporous structure, which effectively inhibited molecular rearrangement and crystallization.

To investigate changes in dissolution performance under accelerated conditions, in vitro dissolution experiments were conducted at various time points (Figure 8D–F). During the 6-month accelerated test period, the 60-min cumulative dissolution of CEL-DMSN remained above 90.0%, decreasing slightly from an initial 96.8% to 93.4% (a drop of only 3.4%), indicating stable dissolution performance. For A-CEL, the 60-min cumulative dissolution dropped from 92.5% to 85.2% (a 7.3% decrease), indicating some reduction in dissolution. For CEL-PVP K30, dissolution behavior was significantly affected by drug crystallization, with the 60-min cumulative dissolution dropping substantially from 91.2% to 75.1% (a 16.1% decrease). In summary, CEL-DMSN effectively maintained excellent in vitro dissolution performance under accelerated conditions, demonstrating stability significantly superior to that of A-CEL and CEL-PVP K30.

## 4. Conclusions

In this study, we successfully designed and synthesized a DMSN carrier characterized by a unique radially aligned pore structure and significant pH-responsive degradation properties. Systematic comparison with seven commercial excipients showed that DMSN exhibited the highest drug loading (39.72%), which correlated with its high specific surface area and pore volume. DSC and XRD analyses confirmed that the carrier effectively stabilized the drug in an amorphous state; this stabilization effect, synergistically enhanced by the superior hydrophilic properties of carriers, resulted in a pronounced acceleration of drug dissolution kinetics. In vitro and in vivo experiments have shown that the pH-responsive degradation behavior of the carrier may be related to the improvement of drug release kinetics and the reduction of gastric mucosal irritation. The oral bioavailability of the drug-loaded system was 1.29 times higher than that of commercial celecoxib capsules. Furthermore, accelerated stability tests revealed that the system exhibited long-term stability—maintaining both the amorphous state and dissolution behavior—far superior to that of the pure amorphous drug or traditional polymeric carriers. In summary, the DMSN carrier developed in this study addresses several limitations of conventional solid dispersion systems by combining high drug loading, rapid dissolution, and improved physical stability. This work not only provided an optimization strategy for CEL delivery but also offered a valuable reference model for the design of novel carriers targeting BCS Class II drugs.

## Figures and Tables

**Table 1:** Particle sizes and N_2_ adsorption characteristics of commercial excipients and DMSN.

	Syloid^®^ XDP3050	Syloid^®^ 244 FP	PVP K90	PVP K30	PEG6000	PEG4000	TPGS	DMSN
**Particle size** **(µm)**	37.82	6.00	82.15	81.39	0.238	0.236	37.59	0.200
**S_BET_ (m^2^/g)**	407.80	428.00	1.43	0.24	0.15	0.31	0.074	649.87
**V_P_ (cm^3^/g)**	1.70	1.70	0.0048	0.0004	0.0003	0.0010	0.0001	3.49
**W_BJH_ (nm)**	20.00	17.00	7.67	8.57	3.61	4.22	2.61	21.51

**Table 2:** N_2_ adsorption–desorption parameters and drug loading efficacy of drug loading systems.

Samples	S_BET_ (m^2^/g)	V_t_ (cm^3^/g)	D_p_ (nm)	Drug Loading (%)
	Before/After Drug Loading			
CEL-DMSN	626.08/198.14	3.54/1.39	19.58/19.61	39.72 ± 1.23
CEL-Syloid^®^XDP3050	407.85/151.15	1.74/0.59	20.03/10.76	16.57 ± 0.45
CEL-PVP K30	0.24/0.58	0.0048/0.0031	7.67/9.77	32.75 ± 2.67
CEL-PVP K90	1.43/0.352	0.0048/0.0077	7.67/6.12	33.12 ± 1.89
CEL-Syloid^®^244 FP	428.00/248.35	1.70/1.42	20.02/16.12	19.75 ± 0.97
CEL-TPGS	0.074/0.0232	0.0001/0.0001	2.61/1.03	10.77 ± 2.34
CEL-PEG6000	0.15/0.034	0.0003/0.0001	3.61/1.22	16.64 ± 0.56
CEL-PEG4000	0.31/0.14	0.0010/0.0005	4.22/2.07	16.54 ± 1.75

**Table 3:** Angles of repose data for drug delivery systems (n = 3).

Samples	Angle of Repose (°)	Contact Angle (°)
CEL	/	90.25 ± 0.47
CEL-DMSN	29.64 ± 1.03	15.33 ± 0.24
CEL-Syloid^®^XDP3050	28.54 ± 0.26	16.14 ± 0.33
CEL-PVP K30	23.28 ± 0.47	10.24 ± 0.35
CEL-PVP K90	24.37 ± 0.56	9.15 ± 0.64
CEL-Syloid^®^244 FP	29.32 ± 0.43	23.41 ± 0.16
CEL-TPGS	22.42 ± 0.25	17.26 ± 0.59
CEL-PEG6000	24.32 ± 0.72	10.34 ± 0.24

**Table 4:** Comparison of pharmacokinetic parameters of CEL in rats after oral administration (Mean ± SD, n = 5).

Formulation	T_max_ (h)	C_max_ (μg/mL)	AUC_0~48h_ (μg/mL·h)	Fr (%)
CEL-DMSN	1.92 ± 0.61	6.16 ± 1.47	27.05 ± 6.77	128.9
CEL-PVP K30	2.75 ± 1.08	3.14 ± 0.88	25.04 ± 2.60	119.4
Commercial capsules	2.20 ± 0.93	3.91 ± 1.25	20.98 ± 3.74	/

## Data Availability

The original contributions presented in this study are included in the article/Supplementary Material. Further inquiries can be directed to the corresponding author.

## Supplementary Materials

The following supporting information can be downloaded at: https://www.mdpi.com/article/10.3390/pharmaceutics18030311/s1. Figure S1: UV-scanning spectra of CLB, DMSN and seven commercial solid dispersion excipients in ethanol solution; Figure S2: The standard curve of CEL in ethanol.; Figure S3: Typical HPLC chromatograms of CEL in rat plasma: (A) blank plasma (BP); Figure S4: Standard curve of CEL in plasma; Figure S5: SEM of CEL-DMSN after 6 months of storage (Scale bar = 200 µm). Table S1: Precision experiment of CEL for content determination in ethanol (n = 3); Table S2: The recovery of CEL determined by UV method in ethanol (n = 3); Table S3: Precision for determination of CEL in plasma (n = 5); Table S4: Extraction recovery of CEL in plasma (n = 3); Table S5: Assessment of content uniformity under accelerated conditions.
