# Supplementary material for: Preliminary Study on Different Types of Solid Dispersion Excipients for Improving the Water Solubility and Physical Stability of Celecoxib
Source: Pharmaceutics. 2026 Feb 28;18(3):311. doi: 10.3390/pharmaceutics18030311 (PMC13029672; doi:10.3390/pharmaceutics18030311)
Supplement: Supplementary file 1 [file pharmaceutics-18-00311-s001.zip › pharmaceutics-4131159-supplementary.pdf]

Supporting Information

Preliminary Study on Different Types of Solid Dispersion Excipients for Improving the Water Solubility and Physical Stability of Celecoxib

Bin Liu [1,†], Shiqiao Rui [1,†], Yupan Cai [1], Ruoru Qian [2], Shuaipeng Feng [1], Zhu Liu [1] and Qinfu Zhao [1,*]

[1] Department of Pharmaceutics, School of Pharmacy, Shenyang Pharmaceutical University, 103 Wenhua Road, Shenyang 110016, China; 18341433302@163.com (B.L.); m18611538683_1@163.com (S.R.); gong1234561128@163.com (Y.C.); 13478025275@163.com (S.F.); liuzhu0899@163.com (Z.L.)

[2] Changzhou Pharmaceutical Factory Co., Ltd., 518 East Laodong Road, Tianning District, Changzhou, China; 13940107910@163.com

* Correspondence: qinfuzhao@syphu.edu.cn; Tel.: +86-24-43520537

† These authors contributed equally to this work.

1. Methods

1.1 Establishment of *in vitro* analysis methods

1.1.1 Selection of measurement wavelength

Determination of CEL UV absorption wavelength: Weigh an appropriate amount of CEL and place it in a beaker, add an appropriate amount of ethanol, sonicate and transfer it to a 50 mL volumetric flask, dilute with ethanol, and prepare a 10 μg/mL CEL ethanol solution. Take an appropriate amount of DMSN and seven commercial solid dispersion excipients (PVP K30, PVP K90, TPGS, PEG4000, PEG6000, Syloid®XDP3050, and Syloid®244 FP)， A mixed solution with a concentration of 10 μg/mL was prepared using ethanol as the solvent, and the filtrate was collected after filtration through a 0.45 μm microporous membrane. Scan CEL, DMSN, and seven commercial solid dispersion excipients solutions in the wavelength range of 200-400 nm using ethanol as a blank control.

1.1.2 Investigation of linear relationship

Preparation of the calibration curve for CEL content determination: 20.20 mg of CEL was accurately weighed and placed in a 100 mL volumetric flask. Ethanol was added to dissolve the substance, and the volume was made up to the mark. The solution was shaken well to prepare a stock solution with a concentration of 202.0 μg/mL. Appropriate volumes of the CEL stock solution were diluted with ethanol to prepare a series of standard solutions with concentrations of 4.04 μg/mL, 6.06 μg/mL, 8.08 μg/mL, 10.1 μg/mL, 12.12 μg/mL, 14.14 μg/mL, and 16.16 μg/mL. Using ethanol as the blank control, the absorbance of the series of standard solutions was measured at 242 nm. A calibration curve was constructed by plotting the absorbance on the ordinate against the concentration of CEL on the abscissa.

1.1.3 Precision experiment

Appropriate volumes of the CEL stock solution were accurately measured and diluted with ethanol to prepare CEL solutions at concentrations of 6.06 μg/mL, 12.12 μg/mL, and 16.16 μg/mL. Each concentration solution was prepared in triplicate. Using ethanol as the blank, the absorbance of each working solution was measured at 242 nm. Each solution was measured three times in parallel, and the intra-day precision (RSD) was calculated. The same procedure was repeated for three consecutive days to determine the inter-day

precision (RSD).

1.1.4 Recovery investigation

Appropriate volumes of the CEL stock solution were accurately measured and diluted with ethanol to prepare CEL solutions at concentrations of 6.06 μg/mL, 12.12 μg/mL, and 16.16 μg/mL. Each concentration solution was prepared in triplicate. Using ethanol as the blank, the absorbance of each working solution was measured at 242 nm. Each solution was measured three times in parallel, and the recovery was calculated.

1.2 Establishment of *in vivo* pharmacokinetic analytical method

1.2.1 Chromatographic conditions

Chromatographic column: Diamonsil C18 column (250 mm × 4.60 mm, 5 μm)

Mobile phase: Methanol-water (75:25, v/v)

Detection wavelength: 320 nm

Column temperature: 35°C

Flow rate: 1.00 mL/min

Injection volume: 20.00 μL

1.2.2 Preparation of standard solution

10.01 mg of CEL was accurately weighed, placed in a beaker, and dissolved with an appropriate amount of methanol by ultrasonication. The solution was transferred to a 100 mL volumetric flask, diluted to the mark with methanol, and shaken well to obtain a CEL stock solution with a concentration of 100.10 μg/mL.

1.2.3 Treatment method for plasma samples

400 μL of rat plasma sample was precisely aspirated, and 1.5 mL of methanol was added. The mixture was vortexed for 3 min to ensure homogeneity and then centrifuged (3000 rpm, 10 min). After filtration through a 0.22 μm membrane, the supernatant was collected and analyzed by injection under the chromatographic conditions described in section "2.1.1".

1.2.4 Method specificity

Blank plasma, blank plasma spiked with CEL working standard (final concentration 3.00 μg/mL), and rat plasma samples collected after administration were processed according to the method described in section "2.1.3" and injected for determination under the chromatographic conditions specified in section "2.1.1". The chromatograms were

recorded.

1.2.5 Preparation of calibration curve

Accurately measure an appropriate volume of the 100.10 μg/mL CEL stock solution prepared in Section "2.1.2". Using 200.00 μL of blank plasma as the matrix, add the CEL stock solution and 400.00 μL of methanol successively. Vortex the mixture to obtain plasma samples with CEL concentrations of 0.20, 1.00, 2.00, 4.00, 5.00, 7.00, 9.00, and 10.00 μg/mL, respectively. After processing according to the method in section "2.1.3", the peak areas were recorded. Linear regression analysis was performed by plotting the peak area (Y) on the ordinate against the CEL concentration (C, μg/mL) on the abscissa to obtain the calibration curve equation.

1.2.6 Method precision

Blank plasma (100 μL) was taken, and appropriate amounts of the CEL stock solution from section "2.1.2" were added to prepare plasma samples at low, medium, and high concentrations (1.00, 5.00, and 9.00 μg/mL). Each concentration was prepared in triplicate. The samples were processed according to the method described in section "2.1.3" and then injected for determination under the chromatographic conditions specified in section "2.1.1". The peak areas were recorded, and the concentrations were calculated to determine the intra-day precision (RSD). The same procedure was repeated for three consecutive days to calculate the inter-day precision (RSD).

1.2.7 Extraction recovery

The CEL standard solutions at low, medium, and high concentrations (1.00, 5.00, and 9.00 μg/mL) from section "2.1.6" were accurately measured and injected for determination under the chromatographic conditions specified in section "2.1.1", and the peak areas were recorded (A_1). Separately, appropriate amounts of the CEL stock solution were precisely added to 100 μL of blank plasma to prepare plasma samples corresponding to the low, medium, and high concentrations. These samples were processed according to the method described in section "2.1.3" and then injected for determination, and the peak areas were recorded (A_2). The extraction recovery and RSD value for each concentration were calculated to evaluate the reliability of the plasma sample pretreatment method. The extraction recovery was calculated according to the formula:

$$\text{Extraction recovery (\%)}=\frac{A_2}{A_1}\times 100\%$$

In our study, the relative bioavailability (Fr) was calculated using the standard formula based on the area under the plasma concentration-time curve (AUC). Specifically, Fr was determined as follows:

$$Fr(\%)=\left(\frac{AUC_{0\rightarrow 48h(test)}}{AUC_{0\rightarrow 48h(reference)}}\right)\times 100$$

$AUC_{0\rightarrow 48h(test)}$ represents the area under the plasma concentration-time curve from 0 to 48 hours for the test formulations (CEL-DMSN or CEL-PVP K30)

$AUC_{0\rightarrow 48h(reference)}$ represents the corresponding AUC value for the reference formulation (commercial capsules)

1.3 Animals

Male Sprague-Dawley (SD) rats (180-220 g, 7-9 weeks old). were purchased from Liaoning Changsheng Biotechnology Co., Ltd. After a two-week acclimation period, they were maintained under standard conditions and had free access to a standard diet and water. All procedures adhered to animal care guidelines and were approved by the Animal Ethics Committee of Shenyang Pharmaceutical University (Approval No.: SYPU-IACUC-S2025-1015-109).

2. Results

2.1 Establishment of *in vitro* analytical methods

2.1.1 Selection of determination wavelength

The UV absorption spectrum is shown in Figure S1. The results indicated that CEL exhibited maximum absorption at the wavelength of 242 nm, while DMSN and the seven commercial solid dispersion excipients showed no UV absorption at this wavelength, thus posing no interference with the content determination of CEL. Therefore, 242 nm was selected as the determination wavelength for CEL.

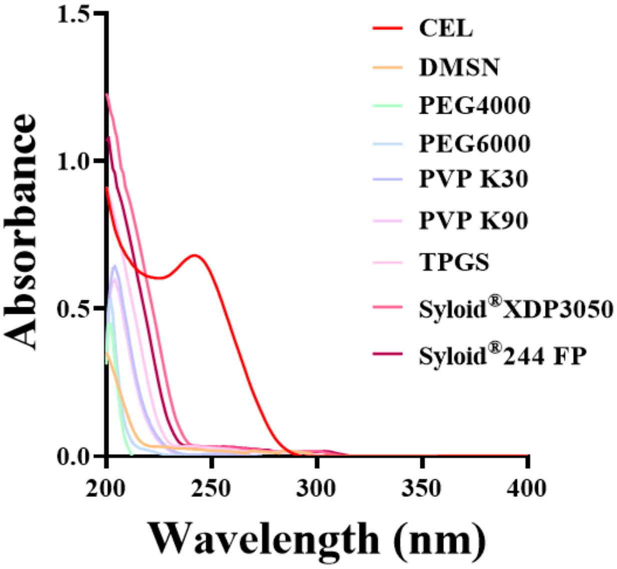

Figure S1 UV-scanning spectra of CLB, DMSN and seven commercial solid dispersion excipients in ethanol solution.

2.1.2 Investigation of linear relationship

Using the concentration of CEL (C) as the abscissa and the absorbance (A) as the ordinate, the calibration curve equation for CEL content determination was obtained as A = 0.0534C - 0.016 (r = 0.9998). The curve is shown in Figure S2. The results demonstrated that, in ethanol solution, the concentration of CEL exhibited a good linear relationship with absorbance within the range of 4.04–16.16 μg/mL, meeting the requirements for quantitative analysis.

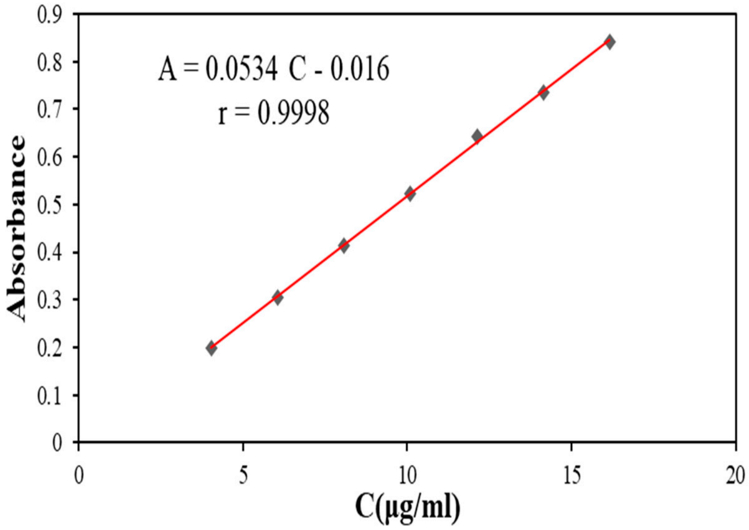

Figure S2 The standard curve of CEL in ethanol.

2.1.3 Precision experiment

The results of the precision experiment for CEL in ethanol are shown in Table S1. The results indicated that the RSD values for both intra-day and inter-day precision were less than 2.00%, indicating good precision of the method and meeting the methodological requirements.

Table S1 Precision experiment of CEL for content determination in ethanol (n = 3)

Concentration	Within-day		Between-day	
(μg/mL)	Mean (μg/mL)	RSD (%)	Mean (μg/mL)	RSD (%)
4.04	4.07	0.25	4.02	0.31
10.10	10.14	0.19	10.07	0.16
16.16	16.21	0.31	16.14	0.26

2.1.4 Recovery investigation

The recovery of samples at low, medium, and high concentrations was investigated, and the results are shown in Tables S2, respectively. The results revealed that the recoveries at all concentration points were within the range of 98.0% to 102.0%, with RSD values less than 2.00%, indicating good accuracy of the method and meeting the requirements for methodological validation.

Table S2 The recovery of CEL determined by UV method in ethanol (n = 3)

Added (μg/mL)	Found (μg/mL)	Recovery (%)	Average (%)	RSD (%)
	4.03	99.8		
4.04	4.00	99.0	99.4	0.41
	4.01	99.3		
10.10	10.02	99.2	99.4	0.20

	10.06	99.6		
	10.04	99.4		
	16.03	99.2		
16.16	16.11	99.7	99.5	0.25
	16.08	99.5		

2.2 Establishment of an *in vivo* pharmacokinetic analytical method

2.2.1 Method specificity

The chromatograms of plasma samples are shown in Figure S3. The results indicated that the response of endogenous substances in blank plasma was primarily concentrated before 2 min, with no significant interfering peaks near the CEL chromatographic peak. The retention time of the CEL working standard in plasma was approximately 4–5 min, the peak shape was symmetrical, and the resolution from adjacent peaks was good. In the rat plasma samples collected after administration, the CEL chromatographic peak was completely separated from the endogenous substance peaks, with no interfering impurity peaks. These results demonstrated that the established HPLC method exhibited good specificity and could be used for the accurate determination of CEL concentrations in rat plasma.

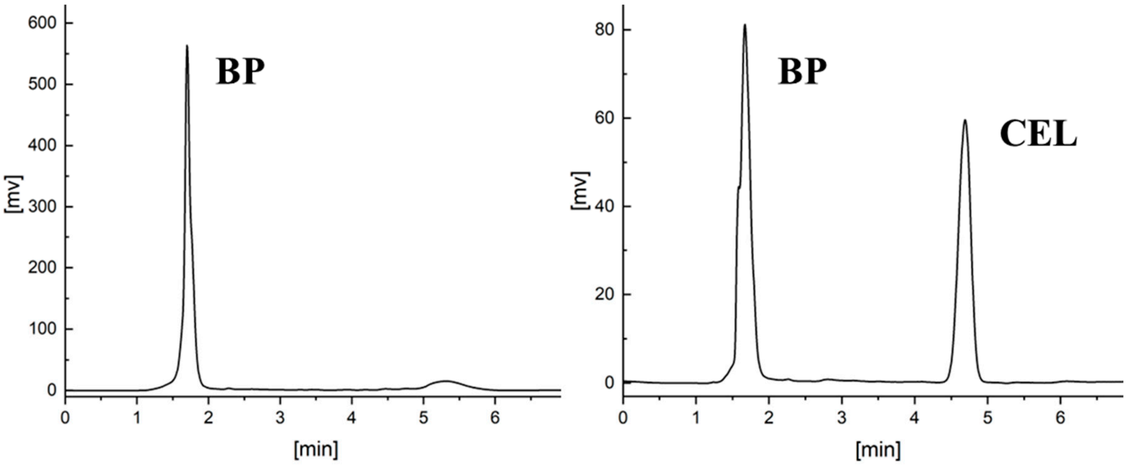

Figure S3 Typical HPLC chromatograms of CEL in rat plasma: (A) blank plasma (BP),

(B) plasma sample after oral administration of CEL-DMSN (2:3).

2.2.2 Preparation of calibration curve

Linear regression was performed by plotting the peak area (Y) on the ordinate against the CEL concentration in plasma (C, μg/mL) on the abscissa. The calibration curve equation obtained was Y = 5520.7C + 1515.9, with a correlation coefficient (r) of 0.9997. The result is shown in Figure S4. This indicated that CEL exhibited a good linear relationship with the peak area within the concentration range of 0.20–10.00 μg/mL, meeting the requirements for the quantitative analysis of plasma samples.

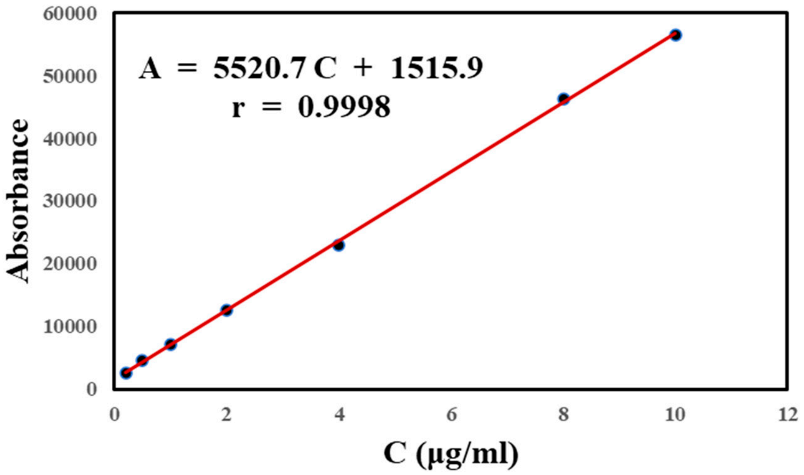

Figure S4 Standard curve of CEL in plasma.

2.2.3 Method precision

The results of the precision experiment for CEL plasma samples are shown in Table S3. The results showed that the RSD values for both intra-day and inter-day precision at low, medium, and high concentrations were less than 10.0%, indicating that the method exhibited good precision. This met the requirements for the quantitative analysis of biological samples and confirmed the method's suitability for determining CEL concentrations in rat plasma.

Table S3 Precision for determination of CEL in plasma (n = 5)

Concentration	Within-day		Between-day	
(μg/mL)	Mean (μg/mL)	RSD (%)	Mean (μg/mL)	RSD (%)
1.00	0.98	3.85	1.02	4.20
5.00	4.95	2.56	5.06	2.95

9.00	8.93	1.73	9.08	2.10

2.2.4 Extraction recovery

The results of the extraction recovery determination are shown in Table S4. The results indicated that the extraction recoveries at low, medium, and high concentrations were all within the range of 85.00%–115.00%, with RSD values less than 10.00%, meeting the requirements for the quantitative analysis of biological samples. This demonstrated that the plasma sample pretreatment method was stable and reliable, with minimal random error during the extraction process, ensuring the reproducibility of drug extraction.

Table S4 Extraction recovery of CEL in plasma (n = 3)

Added (μg/mL)	Found (μg/mL)	Recovery (%)	Average (%)	RSD (%)
	0.86	86.0		
1.00	0.92	91.7	90.8	4.5
	0.95	94.8		
	4.66	93.8		
5.00	4.59	91.8	91.1	3.2
	4.38	87.6		
	8.69	96.5		
9.00	8.53	94.8	94.2	2.7
	8.22	91.3		

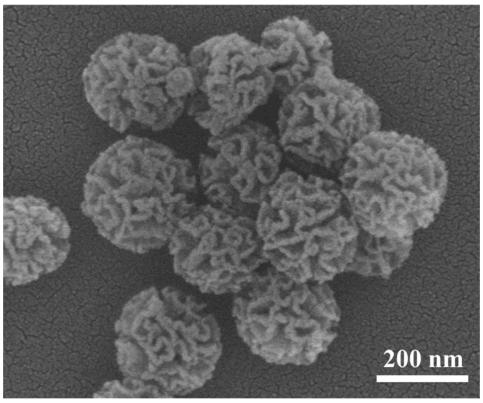

Figure S5 SEM of CEL-DMSN after 6 months of storage (Scale bar = 200 μm).

Table S5. Assessment of content uniformity under accelerated conditions.

Time (months)	X_i (%)		
	CEL-DMSN	CEL-PVP K30	A-CEL
0	100.2 ± 0.20	100.0 ± 0.35	99.9 ± 0.42
1	100.1 ± 0.63	99.3 ± 0.80	95.6 ± 1.23
2	99.8 ± 0.42	98.5 ±0.25	96.7 ± 0.57
3	99.7 ± 0.87	98.2 ± 1.45	97.5 ± 0.89
6	98.5 ± 1.05	94.1 ± 1.66	96.2 ± 1.55
